# Exercise Attenuates Renal Dysfunction with Preservation of Myocardial Function in Chronic Kidney Disease

**DOI:** 10.1371/journal.pone.0055363

**Published:** 2013-02-07

**Authors:** Rafael da Silva Luiz, Kleiton Augusto Santos Silva, Rodolfo Rosseto Rampaso, Ednei Luiz Antônio, Jairo Montemor, Danilo Sales Bocalini, Leonardo dos Santos, Luiz Moura, Paulo José Ferreira Tucci, Nayda Parísio de Abreu, Nestor Schor

**Affiliations:** 1 Nephrology Division, Department of Medicine, Federal University of São Paulo (UNIFESP/EPM), São Paulo, Brazil; 2 Cardio-Physiology and Pathophysiology Laboratory, Federal University of São Paulo, São Paulo, Brazil; 3 Laboratory of Genetics and Molecular Cardiology, Heart Institute (InCor), University of São Paulo Medical School, São Paulo, Brazil; The University of Manchester, United Kingdom

## Abstract

Previous studies have suggested that exercise improves renal and cardiac functions in patients with chronic kidney disease. The aim of this study was to evaluate the effects of long-term aerobic swimming exercise with overload on renal and cardiac function in rats with 5/6 nefrectomy (5/6Nx). Eight Wistar rats were placed into 4 groups: Control (C), Control+Exercise (E), Sedentary 5/6Nx (NxS) and 5/6Nx+Exercise (NxE). The rats were subjected to swimming exercise sessions with overload for 30 min five days per week for five weeks. Exercise reduced the effect of 5/6Nx on creatinine clearance compared to the NxS group. In addition, exercise minimized the increase in mean proteinuria compared to the NxS group (96.9±10.0 vs. 51.4±9.9 mg/24 h; p<0.05). Blood pressure was higher in the NxS and NxE groups compared to the C and E groups (216±4 and 178±3 vs. 123±2 and 124±2 mm Hg, p<0.05). In the 200 glomeruli that were evaluated, the NxS group had a higher sclerosis index than did the NxE group (16% vs. 2%, p<0.05). Echocardiography demonstrated a higher anterior wall of the left ventricle (LV) in diastole in the NxS group compared with the C, E and NxE groups. The NxS group also had a higher LV posterior wall in diastole and systole compared with the E group. The developed isometric tension in Lmax of the heart papillary muscle was lower in the NxS group compared with the C, E and NxE groups. These results suggested that exercise in 5/6Nx animals might reduce the progression of renal disease and lessen the cardiovascular impact of a reduction in renal mass.

## Introduction

Kidney disease has a significant impact on patient morbimortality. The pathophysiological spectrum of kidney diseases is broad [1]. Chronic kidney disease (CKD) is characterized by a progressive loss of nephrons, which is caused by increases in intraglomerular pressure and hyperfiltration [2].

CKD leads to reduced physical activity and an increased risk of cardiovascular disease (CVD). A sedentary lifestyle increases the risk of CVD, but CVD can be ameliorated by physical fitness [1], [2], [3].

Aerobic exercise has been shown to improve renal and cardiac function in individuals with CKD, and exercise has gained more attention as a possible tool for preventing, reducing or delaying CKD progression [1], [4], [5], [6]. It has been suggested that appropriate exercise may improve a patient’s physical strength and quality of life [1], [2], [3], [4], [6], [7], [8], [9]. Swimming has been increasingly prescribed as a non-pharmacological treatment for arterial hypertension, obesity and coronary heart disease. Thus, improving our knowledge of the effects of swimming training on cardiovascular function is relevant for CKD patients [10].

Because of the increase in CKD incidence and its associated cardiovascular risks and damage in recent decades, it is important to evaluate the effects of exercise with overload on renal and cardiovascular functions.

## Materials and Methods

### Animals

All of the experimental procedures were conducted according to the National Institutes of Health guidelines for the use and care of animals, and the study protocol was approved by the Ethics in Research Committee at the Federal University of São Paulo (UNIFESP) (process No. 1163/08). Thirty-two male Wistar rats (230–250 g) were obtained from the animal care facility at our institution. The animals were group housed, given access to rat chow and water ad libitum and maintained in a temperature-controlled environment (23°C) on a 12-hour light/dark cycle.

### Experimental Protocols

After a 7-day adaptation period, the animals were weighed and housed in metabolic cages and kept in a humidity and temperature-controlled room for 24 h to collect urine. The following day, the rats were randomly submitted to a five-sixths nephrectomy (5/6Nx) under anesthesia with ketamine (100 mg/kg i.p.) plus xylazine (10 mg/kg i.p.). After ventral laparotomy, removal of the right kidney and ligation of 2 branches of the left renal artery were performed, and infarction of two-thirds of the left kidney was achieved.

One week after the operation, the baseline measurements of body weight (BW) were recorded. The rats were divided into the following four groups and assessed 8 weeks later: sedentary control (C, n = 8), exercise control (E, n = 8), sedentary 5/6Nx (NxS, n = 8) and exercise 5/6Nx (NxE n = 8).

### Exercise Training

The rats went through an initial 7-day adjustment period that consisted of 30 min of swimming in thermo-neutral water (32°C) in a 500 L swimming pool 126-cm in diameter and 63-cm deep. After the initial adjustment period, the animals were subjected to a maximal workload test in the same swimming pool at the same water temperature. For this test, the animals were placed individually in the tank with a workload that increased at intervals of 3 min with weights corresponding to 1, 2 and 3% of the animal’s body weight. Fishing weights were attached to the tail of the animal until it reached its maximum load, as determined by the time to exhaustion.

The criterion for characterizing exhaustion in the swim test was the inability of the animal to continue swimming and sink without returning to the surface after 10 seconds.

After completing the stress test, the training regimen was established by maximal workload using a rule of three (100% = maximal workload and X = 70%). Two weeks post-surgery (including one week of water adaptation), the animals underwent a 5-week exercise protocol at 70% of the maximal workload, totaling 7 weeks of 5/6NX by the end of the protocol. This protocol was adapted from Osorio et al. [11].

### Renal Function

Labtest Diagnostic Kits® were used to obtain the creatinine levels using the picrate method, which is closely related to the methods using Lloyd’s reagent or ion exchange chromatography. Proteinuria was measured using an enzymatic colorimetric assay from a commercial kit (Sensiprot, Labtest Diagnostic) [3].

### Systolic Blood Pressure

Systolic blood pressure (SBP) was monitored in the conscious rats using the tail cuff method (PE300; Narco Bio-Systems) at the beginning and end of the experiment [12].

### Renal Histology

After the functional analysis of the heart, the remaining kidney was removed and placed in sterile phosphate buffer at 4°C and sectioned with a scalpel into fragments for the morphological study. These fragments were then fixed in 10% buffered formalin and processed according to standard techniques for paraffin embedding. Then, 3-µm-thick sections were placed on glass slides and stained with hematoxylin-eosin, periodic acid-Schiff (PAS) and Masson Trichrome. To determine the degree of glomerular sclerosis, the samples were examined by a single pathologist, who was blinded to the renal status of the groups. To establish the degree of glomerular injury, all of the glomeruli were counted in each section that was stained with PAS. This total was used to determine the number of glomeruli that showed sclerosis, characterized by mesangial matrix expansion and obliteration of capillary lumens synechiae of glomerulus adherent to Bowman’s capsule. To calculate focal glomerular sclerosis, 200 glomeruli from each stained section were examined [1].

### Echocardiography

After ketamine*-*xylazine anesthesia (i.p.), transthoracic echocardiography was performed by an observer blinded to the animal’s group, as previously described [13], using an HP Sonos-5500 echocardiograph (Hewlett Packard, Andover, MA, USA) with a 12-MHz linear transducer. The rats were imaged in the left lateral decubitus position with three electrodes placed on their paws for the electrocardiogram. Two-dimensional parasternal long- and short-axis views and 2D-targeted M-mode tracings throughout the anterior and posterior left ventricular (LV) walls were recorded. The anterior (LVAWd) and posterior (LVPWd) LV wall thickness at diastole and systole, the LV end-diastolic (LVEDD) and end-systolic (LVESD) diameters, LV fractional shortening (FS), ejection fraction (EF), E and A wave and E/A relationship were obtained.

### LV Hemodynamics

Immediately after echocardiography, the rats were intubated, ventilated (Rodent Ventilator, Harvard Apparatus Mod 683; Holliston, MA, USA) and a 2-F Millar catheter-tip micromanometer was inserted through the right carotid artery into the LV cavity. Measurements of LV parameters, including LV systolic pressure (LVSP), LV end-diastolic pressure (LVEDP), and maxima positive (+dP/dt) and negative (–dP/dt) time derivatives of the developed pressure, were studied using AcqKnowledge 3.5.7 software (Biopac Systems Inc., Santa Barbara, CA, USA) [14].

### Isolated Papillary Muscle Mechanics

Immediately after the hemodynamic analysis, the heart was quickly removed and placed in an oxygenated Krebs solution. In accordance with other studies performed in our laboratory [10], [13], [15], [16], the papillary muscle was carefully dissected, mounted between two spring clips and placed vertically in a chamber containing Krebs solution (28°C) oxygenated with 100% O_2_, a pH of 7.40±0.02 and a temperature of 30°C. After a 60-min equilibration period during which the preparations allowed isotonic contraction under light loading conditions (0.43 g), papillary muscles were loaded to contract isometrically for 15 min and stretched to the apices of their length–tension curves (Lmax). The mechanical behavior of the papillary muscles was evaluated under basal conditions and at 98, 96, 94 and 92% of Lmax to determine the Frank–Starling curves. The following parameters were measured during isometric contractions: peak developed tension (DT), resting tension (RT), maximum rate of tension development (+dT/dt) and tension decline (–dT/dt), time to peak tension (TPT) and time from peak tension to 50% relaxation (RT50%). At the end of each experiment, the muscle length at Lmax was measured, and the muscle between the two clips was blotted dry and weighed. The muscle cross-sectional area (CSA) was calculated from the muscle weight and length by assuming cylindrical uniformity and a specific gravity of 1.

### Statistical Analysis

The data are expressed as the mean ± SEM. A one-way ANOVA was used to determine the differences among the group means followed by post-hoc Tukey’s tests. Statistical analyses were performed using Prism software (version 5.0, San Diego, CA, USA). Values of p<0.05 were considered statistically significant.

## Results

### Renal Function

The initial renal function data are shown in **Table 1**. After the training period (7 weeks post-surgery), the animals in the NxS and NxE groups had lower body weights (313±2 and 305±1, respectively) than did the animals in group C but not group E (362±9 and 342±7 g, respectively, p<0.05).

**Table 1:** Initial renal function data in the sedentary control (C), exercise control (E), sedentary 5/6Nx (NxS), and exercise 5/6Nx (NxE) groups.

Variables	C	E	NxS	NxE
Body weight (g)	237±3	237±2	246±4	244±2
Scr (mg/dL)	0.35±0.02	0.38±0.03	0.49±0.07	0.37±0.02
CrCl (ml/min)	1.37±0.07	1.22±0.13	0.93±0.10	0.95±0.13
Uprot (mg/24 h)	15.6±1.1	15.75±2.0	18.5±1.8	16.11±1.1
BP (mmHg)	123±2	124±2	120±2	119±2

Results are expressed as the mean ± SEM (n = 8 per group). Serum creatinine (Scr), creatinine clearance (CrCl), proteinuria (Uprot) and blood pressure (BP).

**Figure 1:**
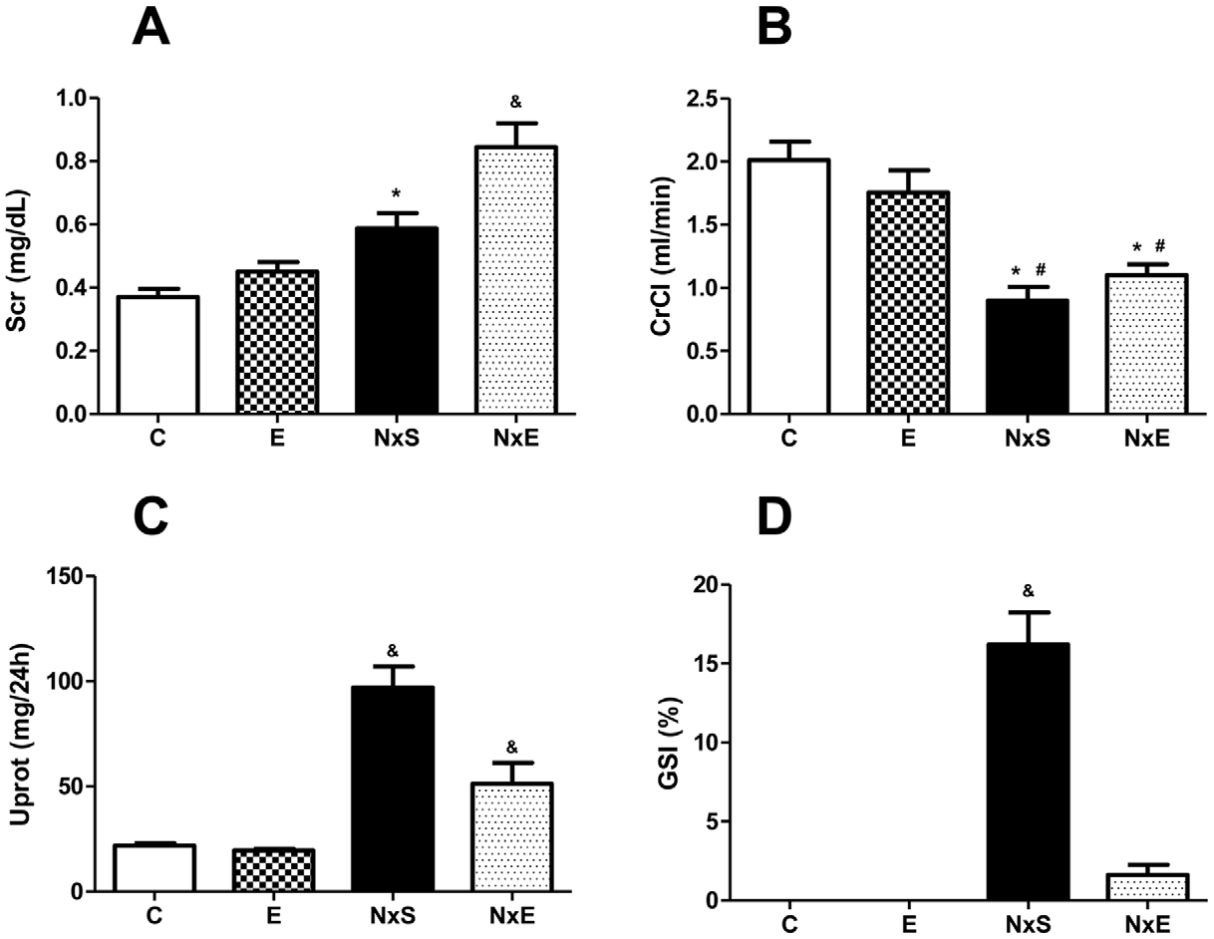
Renal Function. Sedentary control (C), exercise control (E), sedentary 5/6Nx (NxS), exercise 5/6Nx (NxE). **Figure 1** **A** - serum creatinine (Scr) expressed in mg/dL, **Figure 1** **B** - creatinine clearance (CrCl) expressed in ml/min, **Figure 1** **C** - proteinuria (Uprot) expressed in mg/24 h and **Figure 1** **D** – glomerular sclerosis index (GSI) expressed in %. **^&^** p<0.05 vs. all groups.

**Figure 2:**
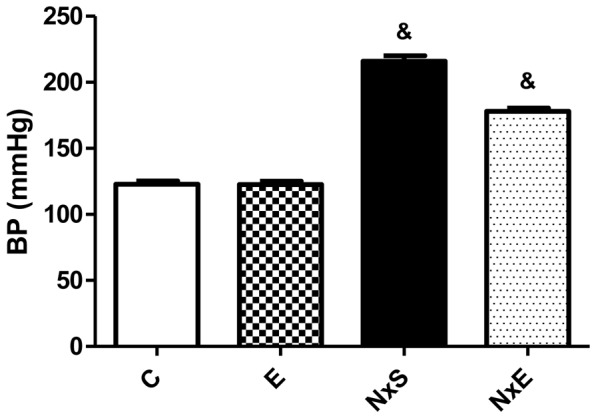
Blood Pressure. BP (Blood Pressure). Sedentary control (C), exercise control (E), sedentary 5/6Nx (NxS), exercise 5/6Nx (NxE). **^&^** p<0.05 vs. all groups.

The mean Scr **(** **Figure 1** **A)** was significantly different between the NxS and NxE groups (0.59±0.05 vs. 0.84±0.07 mg/dl, p<0.05). The creatinine clearance **(** **Figure 1** **B)** also differed between the NxS and the NxE groups relative to the C and the E groups (0.90±0.11 and 1.10±0.09 vs. 2.01±0.15 and 1.76±0.18 ml/min, respectively, p<0.05). Proteinuria **(** **Figure 1** **C)** was higher in the NxS and NxE groups compared to the other groups. However, the NxE group had close to 50% significantly lower values than the NxS group (51.4±9.9 mg/24 h vs. 96.9±10.1 mg/24 h, p<0.05). Blood pressure was higher in the NxS and the NxE groups compared to the C and the E groups (216±4 and 178±3 vs. 123±2 and 124±2 mmHg, respectively, p<0.05). When comparing the NxS group with the NxE group, blood pressure was also significantly different (**Figure 2**). From the 200 glomeruli that were evaluated, the NxS group had a higher index of alterations compared to the NxE group (16% vs. 2%, p<0.05) **(** **Figure 1** **D)**.

### Cardiac Function

The echocardiography (ECHO) revealed a higher left ventricular pressure in diastole in the NxS group compared with the C and E groups (p<0.05). There was also an increase in the anterior LV wall thickness at systole (LVPWs) in the NxS group compared with the C group (p<0.05). The mean anterior LV wall thickness at diastole (LVAWd) was higher for the NxS animals compared with the animals from groups C, E and NxE (p<0.05). No differences were observed in LVAWs among the groups. The basal hemodynamic pressure was similar between the groups (**Table 2**).

**Table 2:** Cardiac function in the sedentary control (C), exercise control (E), sedentary 5/6Nx (NxS), and exercise 5/6Nx (NxE) groups.

	Experimental Groups			
Variables	C	E	NxS	NxE
***Cardiac Biometric***				
LVW/BW (g)	2.10±0.08	2.25±0.06	3.20±0.11&	2.80±0.20* #
CW/BW (g)	2.77±0.09	2.90±0.11	3.94±0.09* #	3.55±0.25* #
Atrium/BW (g)	0.11±0.01	0.13±0.01	0.16±0.01*	0.14±0.01
***Echocardiography***				
LVPWd (cm)	0.10±0.00	0.10±0.00	0.13±0.01* #	0.12±0.00
LVPWs (cm)	0.19±0.01	0.20±0.01	0.23±0.02*	0.22±0.01
LVAWd (cm)	0,10±0,01	0,08±0,00	0,13±0,01&	0,11±0,00#
LVAWs (cm)	0,19±0,01	0,18±0,01	0,20±0,01	0,19±0,01
***Basal Hemodynamics***				
LVSP (mmHg)	112±4	122±2	124±3	113±3
LVEDP (mmHg)	6.0±0.9	6.0±0.9	6.0±1.7	5.3±0.8
+dP/dt (mmHg/sec)	6585±1507	9157±612	7607±670	7089±821
-dP/dt (mmHg/sec)	−5057±226	−5924±181	−5002±348	−4982±309

Results are expressed as the mean ± SEM (n = 8 per group). LVW/BW, left ventricle weight divided by body weight; CW/BW, cardiac weight divided by body weight; LVPWd, diastolic LV posterior wall thickness; LVPWs, systolic LV posterior wall thickness; LVAWd, diastolic LV anterior wall thickness; LVAWs, systolic LV anterior wall thickness; +dP/dt, maximum positive time derivative of developed pressure; –dP/dt, maximum negative derivative of developed pressure.

* p<0.05 vs. C;

# p<0.05 vs. E and

& p<0.05 vs. all groups.

**Figure 3:**
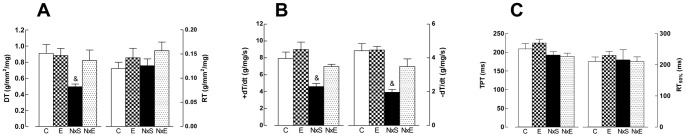
Papillary Muscle. Sedentary control (C), exercise control (E), sedentary 5/6Nx (NxS), exercise 5/6Nx (NxE). **Figure 3** **A** - developed tension (DT), resting tension (RT) expressed in g/mm^2^/mg, **Figure 3** **B** - maximum rate of tension development (+dT/dt) and tension decline (–dT/dt) derivative expressed in g/mg/s and **Figure 3** **C** - time to peak tension (TPT) and time from peak tension to 50% relaxation (RT_50%_) expressed in ms. **^&^** p<0.05 vs. all groups.

For developed isometric tension in the heart papillary muscle (Lmax), the peak developed tension (DT) was lower only in the NxS group compared to the C, E and NxE groups (0.49±0.03 vs. 0.91±0.11, 0.88±0.09 and 0.82±0.13 g/mg, respectively, p<0.05); the resting tension (RT) did not differ among groups (**Figure 3** **A**). The maximum rate of tension development (+dT/dt) was lower in the NxS group vs. the C, E and NxE groups (4.6±0.4 vs. 7.9±0.7, 9.0±0.9 and 7.0±0.3 g/mg/s, respectively, p<0.05). Although the NxS group presented lower values than the C, E and NxE groups, there was only a difference in the -dT/dt (2.0±0.2 vs. 4.4±0.4, 4.5±0.2 and 3.5±0.5 g/mg/s, respectively, p<0.05), as shown in **Figure 3** **B**. The values of contractility did not differ among the groups (**Figure 3** **C**).

Although not shown, the data on the cross-sectional area of the papillary muscles, weight of the right ventricle, LV end-diastolic and systolic dimension, fractional shortening, ejection fraction, A and E wave and E/A relationship did not differ among the groups.

In analyzing the biometric data of the hearts, the 5/6Nx groups had increased left ventricular (LV) weight. The mean for the NxS group was higher compared with the means of groups C, E and NxE (p<0.05).

After the heart weight was corrected for body weight (BW index), the NxS and NxE groups were higher compared to the C and E groups (p<0.05). When the weight of the atrium was corrected for BW (Atrium/BW), only the NxS group was different from the C group; however, significant differences were not observed when the NxS group was compared with the E and NxE groups (**Table 2**).

## Discussion

In this study, the renal and cardiac effects of exercise (EXE) with overload in a rat model of CKD were assessed. Decreases in CrCl and progressive increases in proteinuria with high glomerulosclerosis and blood pressure associated with 5/6Nx in Wistar rats were observed. Sedentary animals with nephrectomy were observed to have dysfunctions in myocardial contractility. The renal function data (Scr, CrCl and proteinuria) are similar to previously reported data [1], [3], [17], [18], [19], [20]. However, no reported data have addressed the association between renal and cardiac function in response to swimming EXE and overload in animals with 5/6Nx. After the training period, it was observed that the mean body weight of the animals with 5/6Nx was lower than the C group. Body weight appears to be directly related to the type of surgery performed, similar to the results from other studies [1], [21]. The increase in Scr in the NxS and NxE groups from progressive renal disease was similar to what has been observed in other studies [1], [3], [21], [22], [23]. The muscle contains approximately 98% of the total amount of creatine in the body, storing 60 to 70% in the form of phosphocreatine and the rest in free creatine. Thus, total muscle mass is the most important determinant of the total creatine pool and creatinine production [24]. In our study, we did not evaluate muscle mass, but it is reasonable to suggest that the greater increase in serum creatinine in the NxE group compared to the NxS group could be attributed to the increase in muscle mass because of EXE, as has been observed in other studies of overload [1], [25]. Slentz et al. [26] have shown that EXE significantly influenced body composition by reducing body fat and increasing lean body mass. Thus, individuals [27] or animals [1] subjected to physical EXE have greater muscle mass and higher levels of serum and urine creatinine. There were no observed differences in CrCl between control groups C and E because training did not alter the glomerular filtration or albumin excretion in the controls, as was the case in our previous observation [3]. A reduction in CrCl in the NxS and the NxE groups was observed, as expected, when compared with the C and the E groups. In fact, 5/6Nx reduced the CrCl, which confirmed the induction of CKD [20], [23], [28]. Although this parameter was not standardized in the NxE group, a ∼20% increase was observed in relation to the NxS group, although it did not reach the level of statistical significance. Although there were no significant differences, increases of 20% may delay treatment for CKD by replacing renal function with dialysis or transplantation. Osato et al. [29] conducted a study of Lewis rats that were treated with adriamycin, which is a model of sclerosing glomerulonephritis with nephrotic syndrome. They showed a decrease in Scr, an increase in GFR and an alleviation of blood pressure and glomerulosclerosis resulting from 2 h/day of swimming for 20 weeks, with relative food restriction in adriamycin-treated rats.

Proteinuria is considered a prognostic factor for progressive renal disease and is the main manifestation of glomerular disease and disability because of alterations in normal permselectivity [30] and increased activation of the renin angiotensin aldosterone system in 5/6Nx [19]. The mediators that play an important role in developing glomerular injury include vasoactive substances, such as AII, nitric oxide (NO), arachidonic acid derivatives and endothelin [30], [31]. In our study, the NxE group, which was subjected to EXE, was significantly protected compared to the NxS group. A decrease in proteinuria (∼ 50%) and a small blood pressure reduction have also been observed in previous studies with other types of aerobic exercises (treadmill and swimming without overload) [1], [17], [18]. Renal protection as a result of EXE can be confirmed by morphological study and a lower glomerular sclerosis index. The NxE group was less impacted compared with the NxS group. The results of these measures are consistent with the data reported for the same experimental 5/6Nx model [1], [3], [12], [17], [22]. The decrease in proteinuria after EXE may be related to the output of nitric oxide [32], prostaglandins and histamine, which all contributed to the pressure decrease after the training period [33]. In our laboratory, Bergamaschi et al. showed that treadmill exercise (65% to 75% vO_2_max) for 30 min, 5 times/week for 60 days in Munich-Wistar rats with 5/6Nx [3] reduced the total glomerular filtration rate and decreased the proteinuria and glomerulosclerosis indices. One possible explanation for these differences could be that the effects of chronic EXE provoked an efferent arteriolar vasodilatation, inducing a reduction in glomerular hydraulic pressure. Many vasoactive agents that are involved in this situation, such as prostaglandins, renin angiotensin, the sympathetic nervous system [3] and activation of the kallikrein-kinin system [34], could participate in this process. In fact, glomerular hypertension is often considered to be one of the main causes of CKD progression [35]. Anderson et al. [36] demonstrated that controlling glomerular hypertension in nephrectomized rats delayed the CKD progression, preserved renal morphology and reduced proteinuria. It was also suggested that after renal mass ablation, CKD progresses because of an increase in both glomerular perfusion and pressure. Considering this observation, the remaining nephrons compensate for the loss of renal mass by hyperfiltering, which leads to glomerular sclerosis.

Heifets et al. [18] have reported that the effect of chronic EXE on renal function in rats with 75% renal ablation caused significantly higher GFR in trained rats compared to sedentary rats, with no change in renal blood flow or the degree of hypertension. Similar to the present study, proteinuria and glomerulosclerosis were reduced in the trained rats compared to the sedentary animals.

Kohzuki et al. [17] demonstrated a protective effect of EXE after 4 weeks, with a reduction in GSI in groups submitted to EXE on a treadmill for 60 min per day, five days per week at 20 meters per minute in spontaneously hypertensive rats with 5/6Nx. Kanazawa et al. [1] conducted a 12-week study with treadmill EXE in Wistar-Kyoto rats that underwent 5/6Nx. The animals were divided into six treatment groups (control, enalapril, moderate EXE, intense EXE, moderate EXE+enalapril and sham rats). The moderate EXE consisted of 60 min per day, 5 days per week at 20 meters per min. However, in this protocol the speed was increased to 28 meters per minute to characterize intense EXE. Both EXE and enalapril blocked the development of hypertension and blunted the increases in proteinuria and in GSI. These results are consistent with the results obtained in our study, although we used a different exercise model and a renin-angiotensin system blocker.

Echocardiographic abnormalities are common in CKD patients [37]. Changes in size, shape and left ventricular function are present in 70–80% of patients on dialysis [38]. The indices used in clinical practice to assess systolic function, which are the EF and FS, are also dependent on both preload and afterload [39]. In the present study, no changes were observed over six weeks in an animal model with 5/6Nx. Hayashi et al. [40] showed that human patients with CKD presented with cardiac and diastolic-filling velocity abnormalities (E and A). In our study, the shorter duration of CKD that we evaluated may explain these differences in the data. We believe that seven weeks of CKD by 5/6 nephrectomy was not enough to provide a significant difference in this analysis. Changes in hemodynamic parameters occur as a consequence of nephron loss and cardiovascular changes [20], [41]. Windt et al. [20] found differences in systolic and diastolic pressures as well as derivatives of contraction and relaxation after ten weeks in animals that underwent surgical reduction of renal mass; these results differed from our results, which employed a different experimental protocol.

Pathological cardiac hypertrophy is recognized in cardiovascular disease and is an independent risk factor for morbidity and mortality [42]. Cardiac hypertrophy is often regarded as the first sign of the cardiac remodeling process and is a predictor for developing arrhythmias, heart failure and sudden death. A hallmark of pathological remodeling is reduced cardiac contractility and alterations in Ca^2+^ kinetic protein and have frequently been observed [43]. Our CKD results and stages of progressive renal disease were not different from the literature [20], [37]. Evangelista et al. [44] conducted a study that involved six different training protocols of swimming, working with variable intensity, frequency, duration and volume of EXE in mice. After the study period, it was observed that cardiac hypertrophy caused by EXE involved training for 90 min, 2 times per day, 5 times per week for 4 weeks with overload. In our study, the training period was not sufficient to cause increases in the cardiac mass of exercised animals but was adequate for ameliorating cardiac remodeling caused by CKD. Although there is a belief that ventricular remodeling results in progressive deterioration of ventricular function, the mechanisms responsible for this phenomenon are not yet fully understood.

The association of CKD with myocardial contractile dysfunction is commonly known, but the contribution of systolic and diastolic dysfunction to heart disease in patients with CKD is unclear [45]. Our results showed for the first time that EXE overload was able to preserve myocardial contractility in CKD. These results were similar to previous studies that have been performed in our laboratory [10], [46] and showed that EXE attenuated contractile dysfunction in different cardiac diseases. Additionally, Sviglerova et al. [47], using the same technical methodology, found impaired DT (developed tension), TPT (time to peak tension) and RT50% (time from peak tension to 50% relaxation) in aged rats with 5/6Nx. In fact, EXE can improve myocardial dysfunction caused by CKD.

Muscle length variations can promote alterations in myocardial contractility, assuming a key role in myocardium performance. An ancillary, but no less interesting, result in our data is related to the Frank–Starling mechanism. This report describes an upward shift of the length–active tension without alterations to the slope of the curves [15]. In addition, our results showed no change in the length–active tension relations slope, which suggested that there was no influence on the Ca^2+^ sensitivity of myofilaments to lengthening (i.e., there was no influence on the Frank–Starling mechanism). Therefore, the data indicate that in animals with CKD that undergo exercise with overload, contractile function is preserved. The values for DT (development tensions) in the NxE are similar to the control and exercise alone groups. As noted in the animals, the C, E and NxE groups differed from the NxS group (0.91±0.11, 0.88±0.09, 0.82±0.13 vs. 0.49±0.03 g/mm^2^/mg, respectively, p<0.05), demonstrating that DT is preserved when the animals are subjected to swimming with overload.

Considering these results, it might be helpful to perform a clinical study to determine whether these patients die from cardiovascular disease, stroke or cardiac death. Thus, it would be interesting to consider the biomarkers of mortality and morbidity by identifying the at-risk patients at different stages of CKD [48] and correlate this risk with exercise, as performed in our protocol.

### Conclusion

These results suggest that despite the mild effect of chronic EXE with a weight overload on CrCl in the NxE group, which was approximately 20% higher than in the NxS animals, there is an important and significant decrease in proteinuria after the training period (50%) and a striking reduction in glomerular sclerosis in the NxE compared with the NxS group. In addition, the mechanical contraction of the papillary muscle can be attenuated with this type of EXE.
